# Phenoxazine-Dibenzothiophene Sulfoximine Emitters Featuring Both Thermally Activated Delayed Fluorescence and Aggregation Induced Emission

**DOI:** 10.3390/molecules26175243

**Published:** 2021-08-29

**Authors:** Yiyu Yang, Ran Xiao, Xiaosong Cao, Zhanxiang Chen, Xialei Lv, Youming Zhang, Shaolong Gong, Yang Zou, Chuluo Yang

**Affiliations:** 1 Shenzhen Key Laboratory of Polymer Science and Technology, College of Materials Science and Engineering, Shenzhen University, Shenzhen 518060, China; 1910342080@email.szu.edu.cn (Y.Y.); xiaoran@szu.edu.cn (R.X.); lvxl0907@163.com (X.L.); yangzou@szu.edu.cn (Y.Z.); clyang@szu.edu.cn (C.Y.); 2 Key Laboratory of Optoelectronic Devices and Systems of Ministry of Education and Guangdong Province, College of Optoelectronic Engineering, Shenzhen University, Shenzhen 518060, China; 3 Hubei Key Lab on Organic and Polymeric Optoelectronic Materials, Department of Chemistry, Wuhan University, Wuhan 430072, China; zhanxiangchen@whu.edu.cn (Z.C.); slgong@whu.edu.cn (S.G.)

**Keywords:** organic light-emitting diodes, thermally activated delayed fluorescence, color-managing acceptor, aggregation induced emission

## Abstract

In this work, we demonstrate dibenzothiophene sulfoximine derivatives as building blocks for constructing emitters featuring both thermally activated delayed fluorescent (TADF) and aggregation-induced emission (AIE) properties, with multiple advantages including high chemical and thermal stability, facile functionalization, as well as tunable electron-accepting ability. A series of phenoxazine-dibenzothiophene sulfoximine structured TADF emitters were successfully synthesized and their photophysical and electroluminescent properties were evaluated. The electroluminescence devices based on these emitters displayed diverse emissions from yellow to orange and reached external quantum efficiencies (EQEs) of 5.8% with 16.7% efficiency roll-off at a high brightness of 1000 cd·m^−2^.

## 1. Introduction

Materials with thermally activated delayed fluorescence (TADF) have been extensively explored since the pioneering research by Adachi et al. [1]. Due to the merits of diverse emission colors, high efficiency, and low cost, TADF molecules are highly potent in next-generation solid lighting and display devices [2,3,4,5,6]. Mechanistically, the theoretical 100% internal quantum efficiency (IQE) of organic light-emitting diodes (OLEDs) based on TADF emitters is enabled by efficient reverse intersystem crossing (RISC) of the triplet excitons to singlet excited states [7,8,9,10,11]. To facilitate up-conversion of triplet excitons, a small energy splitting (Δ*E*_ST_, below 0.2 eV) between singlet (S_1_) and triplet (T_1_) excited states is required [12,13]. The predominant strategy is the spatial separation of the highest occupied molecular orbital (HOMO) and the lowest unoccupied molecular orbital (LUMO) with twisted donor-acceptor (D-A) linkage to effectively reduce the exchange integral of the orbitals [14,15,16,17].

For TADF emitters with prominent intramolecular charge-transfer (ICT) characters, careful selection of electron-donating or -accepting units allows delicate manipulation of emission colors [18,19,20,21,22]. On the other side, improvements in device performance also heavily rely on judicious molecular design guided by structure–property relationship investigations [23,24,25]. Currently, it has been demonstrated by numerous reports that conjugation of nitrogen-containing aromatics (i.e., carbazole [26], dimethylacridane [27], phenoxazine [28,29]) with acceptors such as aza-aromatic rings [15,16,17,18,19], ketones [29,30,31], nitriles [32,33,34], sulfones [35,36,37], boranes [38,39], amides [40], could realize decent device efficiencies. However, the majority of these structural units are chemically inert and not easy to be functionalized, disfavoring color management or further optimization of device performances. 

**Scheme 1:**
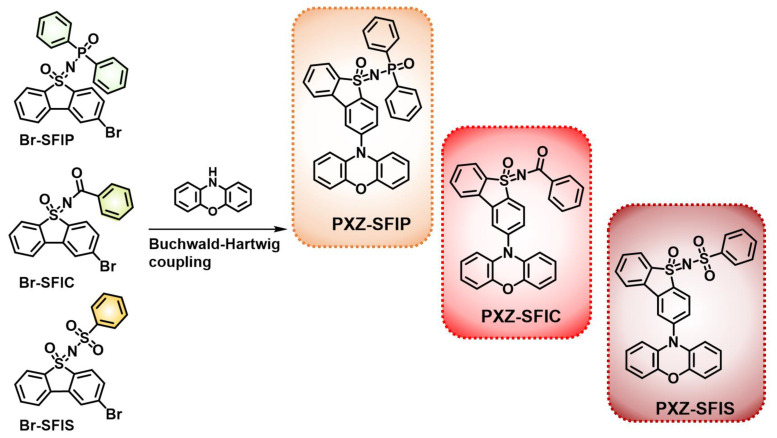
Synthesis route of the TADF emitters with functionalized dibenzothiophene sulfoximine-type acceptor.

Sulfoximines are monoaza analogs of sulfones featuring high stability and versatile chemistry that have been widely studied in the pharmaceutical field [41,42]. Apart from that, the mild basic nitrogen atom in sulfoximine provides the possibility for facile chemical functionalization [43]. In this way, the photophysical properties could be easily adjusted by tuning the electron-withdrawing ability of the functionalized sulfoximines using appropriate *N*-substitutes [44]. In this work, we report three TADF fluorophores with aggregation-induced emission (AIE) character using dibenzothiophene sulfoximine with different *N*-functional units as an acceptor and 10*H*-phenoxazine (PXZ) as a donor. These emitters were denoted as **PXZ-SFIP**, **PXZ-SFIC**, **PXZ-SFIS**, with the *N*H site substituted by diphenylphosphoryl, benzoyl, and benzenesulfonyl groups, respectively, as shown in Scheme 1. The facile metal-free functionalization of dibenzothiophene sulfoximine provides delicate control of electron deficiency of the acceptors and regulates the overall photophysical performance. OLEDs fabricated with these TADF emitters as a dopant in emitting layer obtained diverse electroluminescence (EL) from yellow to orange, with a maximum external quantum efficiency (EQE_max_) exceeding 5%, and insignificant efficiency roll-off of 16.7% at a high brightness of 1000 cd·m^−2^, demonstrating the phenoxazine-dibenzothiophene sulfoximine compounds as promising emitters for TADF-OLEDs.

## 2. Experimental Section

### 2.1. Materials

All starting chemicals and reagents were purchased from commercial suppliers and used without further purification.

### 2.2. General Information

The Fourier-transformed infrared spectra (FT-IR) were recorded on a Thermo Nicolet 6700 Spectrometer. The ^1^H and ^13^C nuclear magnetic resonance (NMR) spectra were collected in dilute CDCl_3_ solution at 25 °C on a Bruker AVANCE III 500 superconducting-magnet high-field NMR spectrometer at 500 MHz and 126 MHz, respectively, using tetramethylsilane (TMS) as an internal standard. Thermogravimetric analysis (TGA) was carried out with a TA instrument TGA Q50 under nitrogen flow (20 mL∙min^−1^), and differential scanning calorimetry (DSC) was performed with a TA DSC Q200 under nitrogen. The glass transition temperature (*T*_g_) was determined from the second heating scan. UV-Vis absorption spectra were recorded on a Shimadzu UV-2700 recording spectrophotometer. Photoluminescence (PL) spectra were recorded on a Hitachi F-4600 fluorescence spectrophotometer. Phosphorescence spectra of doped thin films were conducted at 77 K. The transient photoluminescence decay curves were measured by a single photon counting spectrometer from Edinburgh Instruments (FLS920) with a Picosecond Pulsed UV-LASTER (LASTER377) as the excitation source. The photoluminescence quantum efficiencies were measured using an absolute photoluminescence quantum yield measurement system (C9920-02, Hamamatsu Photonics).

Cyclic voltammetry (CV) was carried out in nitrogen-purged dichloromethane at room temperature with a CHI voltammetric analyzer. Tetrabutylammonium hexafluorophosphate (0.1 M) was used as the supporting electrolyte. The conventional three-electrode configuration consisted of a platinum working electrode, a platinum wire auxiliary electrode, and an Ag/Ag^+^ reference electrode with ferrocenium ferrocene (Fc^+^/Fc) as the internal standard. Cyclic voltammograms were obtained at a scan rate of 100 mV/s. Formal potentials were calculated as the average of cyclic voltammetric anodic and cathodic peaks. The HOMO energy levels of the compounds were calculated according to the formula: −[4.8 + (E_1/2(ox/red)_ − E_1/2(Fc/Fc+_))] eV. The LUMO energy levels of the compounds were calculated according to the HOMO values and the absorption on-set of the longer wavelength. 

### 2.3. Synthesis

General Synthetic Procedures of the TADF Molecules

To a pre-dried 50 mL twin-neck round-bottom flask, Br-SFIP/Br-SFIC/Br-SFIS precursor (1.0 eq), 10*H*-phenoxazine (PXZ, 1.2 eq), palladium acetate (0.1 eq), tri-tert-butylphosphonium tetrafluoroborate (0.2 eq) and cesium carbonate (2.5 eq) were added together with a magnetic stirrer. The flask was evacuated and back-filled with argon for three cycles. Anhydrous toluene (10 mL) was then injected under argon flow via syringe. After refluxing for 36 h, the solvent was evaporated, and then purified by silica gel chromatography (hexanes/ethyl acetate, 8:1 *v/v* in all cases).

#### 2.3.1. **PXZ-SFIP**

Synthesized from compound **Br-SFIP** (0.50 g, 1.01 mmol). 0.57 g, 0.91 mmol yellowish orange solid, 84% yield. FT-IR: *ν*_max_ = 3056, 1588, 1439, 1325, 1244, 1121, 1050, 736, 692, 654, 514 cm^−1^. ^1^H NMR (500 MHz, CDCl_3_) *δ* (ppm): 8.30 (d, *J* = 8.1 Hz, 1H), 8.07 (d, *J* = 7.7 Hz, 1H), 7.89–7.76 (m, 4H), 7.76–7.67 (m, 2H), 7.63 (td, *J* = 7.5, 1.2 Hz, 1H), 7.56 (td, *J* = 7.6, 1.1 Hz, 1H), 7.48–7.28 (m, 7H), 6.77–6.68 (m, 4H), 6.64 (td, *J* = 7.6, 1.9 Hz, 2H), 5.97 (dd, *J* = 8.0, 1.3 Hz, 2H). ^13^C NMR (126 MHz, CDCl_3_) *δ* (ppm): 144.67, 143.98, 139.56 (d, *J* = 3.7 Hz), 138.51 (d, *J* = 2.7 Hz), 135.51 (d, *J* = 132.2 Hz), 135.22 (d, *J* = 132.8 Hz), 135.15, 134.06, 133.32, 133.06, 131.34 (d, *J* = 3.0 Hz), 131.30 (d, *J* = 3.3 Hz), 131.18 (d, *J* = 10.7 Hz), 131.14 (d, *J* = 10.1 Hz), 131.12, 130.68, 128.28 (d, *J* = 3.8 Hz), 128.17 (d, *J* = 3.3 Hz), 126.70, 123.99, 123.61, 123.45, 122.29, 121.82, 115.93, 113.51. 

#### 2.3.2. **PXZ-SFIC**

Synthesized from compound **Br-SFIC** (0.50 g, 1.01 mmol). 0.57 g, 0.91 mmol orange solid, 88% yield. FT-IR: *ν*_max_ = 3058, 1621, 1582, 1482, 1323, 1264, 1217, 1123, 928, 827, 707, 633, 545 cm^−1^. ^1^H NMR (500 MHz, CDCl_3_) *δ* (ppm): 8.64 (d, *J* = 8.2 Hz, 1H), 8.39–8.34 (m, 1H), 8.21–8.12 (m, 2H), 7.86 (d, *J* = 1.8 Hz, 1H), 7.81 (d, *J* = 7.6 Hz, 1H), 7.73 (td, *J* = 7.6, 1.2 Hz, 1H), 7.66 (td, *J* = 7.6, 1.1 Hz, 1H), 7.60 (dd, *J* = 8.1, 1.8 Hz, 1H), 7.54–7.48 (m, 1H), 7.44–7.36 (m, 2H), 6.79–6.69 (m, 4H), 6.65 (td, *J* = 7.6, 1.8 Hz, 2H), 6.06 (dd, *J* = 8.0, 1.4 Hz, 2H). ^13^C NMR (126 MHz, CDCl_3_) *δ* (ppm): 175.22, 145.46, 144.02, 137.55, 136.55, 136.50, 134.84, 134.61, 133.28, 133.25, 132.52, 132.07, 131.31, 129.65, 128.76, 128.12, 125.61, 124.26, 123.48, 122.39, 122.06, 115.99, 113.56.

#### 2.3.3. **PXZ-SFIS**

Synthesized from compound **Br-SFIS** (0.50 g, 1.01 mmol). 0.57 g, 0.91 mmol red solid, 65% yield. FT-IR: *ν*_max_ = 3059, 1582, 1486, 1328, 1312, 1273, 1230, 1150, 1077, 1032, 731, 686, 630, 538 cm^−1^. ^1^H NMR (500 MHz, CDCl_3_) *δ* (ppm): 8.39 (d, *J* = 8.2 Hz, 1H), 8.16 (d, *J* = 7.8 Hz, 1H), 8.13–8.07 (m, 2H), 7.83 (d, *J* = 1.8 Hz, 1H), 7.78 (d, *J* = 7.6 Hz, 1H), 7.72 (td, *J* = 7.6, 1.1 Hz, 1H), 7.65–7.50 (m, 5H), 6.81–6.72 (m, 4H), 6.67 (ddd, *J* = 8.8, 7.1, 2.0 Hz, 2H), 6.04 (dd, *J* = 8.0, 1.3 Hz, 2H). ^13^C NMR (126 MHz, CDCl_3_) *δ* (ppm): 145.99, 144.08, 143.23, 137.13, 136.08, 135.99, 135.06, 133.33, 133.09, 132.54, 131.54, 131.45, 128.85, 127.95, 126.85, 125.18, 124.18, 123.51, 122.56, 122.07, 116.08, 113.63. 

### 2.4. Fabrication and Characterization of Devices 

The ITO coated glass substrates with a sheet resistance of 15 Ω square^−1^ were consecutively ultrasonicated with acetone/ethanol and dried with nitrogen gas flow, followed by 20 min ultraviolet light-ozone (UVO) treatment in a UV-ozone surface processor (PL16 series, Sen Lights Corporation). Then, the sample was transferred to the deposition system. All organic layers were deposited at a rate of 1 Å s^−1^, and subsequently, Liq was deposited at 0.2 Å s^−1^ and then capped with Al (ca. 4 Å s^−1^) through a shadow mask in a vacuum of 2 × 10^−5^ mbar. For all the OLEDs, the emitting areas were determined by the overlap of two electrodes as 0.09 cm^2^. The as-fabricated devices were measured in an ambient environment without any encapsulation. The EL spectra and Commission Internationale de l’Eclairage (CIE) coordinates were recorded with a Keithley 2400 source meter unit. The current density-voltage-luminance (*J*-*V*-*L*) curves of the devices were measured with a PHOTO RESEARCH SpectraScan PR 735 spectrometer. The EQE was calculated from the current density, luminance, and EL (electroluminescence) spectrum, assuming a Lambertian distribution.

## 3. Results and Discussions

### 3.1. Synthesis and Characterization

The Br-SFIP/Br-SFIC/Br-SFIS compounds were prepared according to the previously reported procedure [45]. The donor unit 10*H*-phenoxazine was connected to the Br-SFIX precursor via Buchwald–Hartwig coupling. The ^1^H NMR and ^13^C NMR spectra were in good agreement with the structure of the TADF molecules of **PXZ-SFIP**, **PXZ-SFIC**, and **PXZ-SFIS**. 

### 3.2. Thermal and Electrochemical Properties

Thermogravimetric analysis (TGA) and differential scanning calorimetry (DSC) were employed to investigate the thermal properties of the TADF compounds. High decomposition temperatures (*T*_d_s: corresponding to the temperature at 5% weight loss) of 350, 320, and 355 °C were recorded for **PXZ-SFIP**, **PXZ-SFIC**, and **PXZ-SFIS**, respectively (see ESI, Figure S1). Meanwhile, the emitters presented sufficient high glass transition temperatures (*T*_g_s) to prevent crystallization upon operation of the devices, as indicated by DSC measurements, where **PXZ-SFIC** presented the highest *T*_g_ value of 117 °C, comparing to that of 109 °C for **PXZ-SFIP** and 98 °C for **PXZ-SFIS** (see ESI, Figure S2). To study the electrochemical properties of the designed TADF compounds, cyclic voltammetry was performed (Figure S3). The HOMO levels of the emitters were estimated to be ~−5.19 eV for all cases, reflecting comparable oxidation potential arising from PXZ electron-donating units (Table 1).

### 3.3. Theoretical Calculation

**Figure 1:**
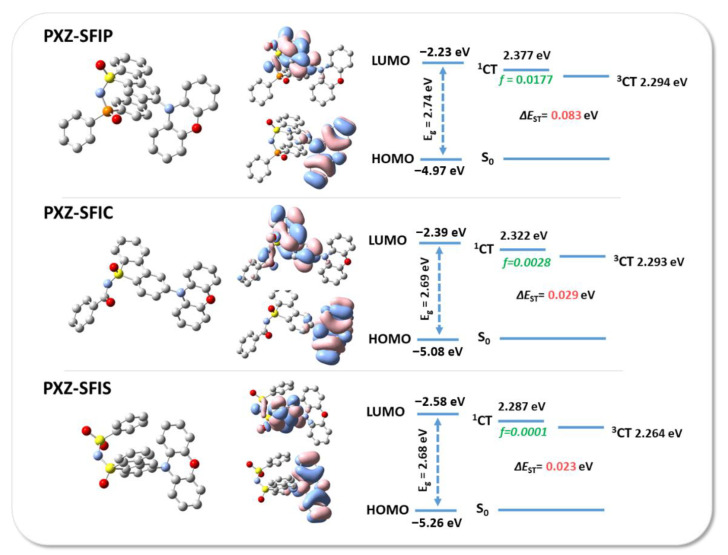
Optimized conformation, frontier orbital distributions, and theoretical calculated energy levels of ground states and excited states of the proposed **PXZ-SFIP**, **PXZ-SFIC**, and **PXZ-SFIS** compounds.

Density functional theory (DFT) calculations at the B3LYP-D3(BJ)/def2-SVP level were carried out to reveal the optimized ground-state geometries and the electronic structures of the designed emitters. As shown in Figure 1, the dihedral angles between the PXZ donor planes and the adjacent dibenzothiophene sulfoximine moieties were measured to be 78.5°, 85.6°, and 85.4° in **PXZ-SFIP**, **PXZ-SFIC,** and **PXZ-SFIS**, respectively, leading to nearly complete separation of the frontier molecular orbitals (FMOs). The diphenylphosphoryl, benzoyl, and benzenesulfonyl substituted dibenzothiophene sulfoximines exhibited a trend of increased electron-withdrawing ability, demonstrated by gradually deepened LUMO levels of −2.23 eV for **PXZ-SFIP**, −2.39 eV for **PXZ-SFIC**, and −2.58 eV for **PXZ-SFIS** in sequence. The energy gaps between HOMO and LUMO of the three compounds were calculated to be 2.74 eV, 2.69 eV, and 2.68 eV, respectively, as depicted in Figure 1. 

The natural transition orbitals (NTO) patterns were investigated to describe the properties of the excited states of the molecules. Time-dependent DFT (TD-DFT) was performed at PBE0-D3(BJ)/def2-SVP level on the basis of the optimized ground-state geometries. Both S_1_ and T_1_ states were typical CT excited states with the ‘hole’ located on the donor moieties, and the ‘particles’ resided on the dibenzothiophene sulfoximines. The S_1_/T_1_ levels of **PXZ-SFIP** (2.38/2.29 eV), **PXZ-SFIC** (2.32/2.29 eV), and those of **PXZ-SFIS** (2.29/2.26 eV) guaranteed relatively small Δ*E*_ST_s for effective thermally activated RISC processes (Table 1). Meanwhile, the oscillator strengths (*f*) from S_0_→S_1_ were calculated to be 0.0177, 0.0028, and 0.0001 for **PXZ-SFIP**, **PXZ-SFIC,** and **PXZ-SFIS**, respectively, predicting a higher singlet radiative transition rate (*k*_r,S_) of **PXZ-SFIP** due to the presence of small yet adequate overlap between its FMOs. The corresponding information is summarized in Table S1.

### 3.4. Photophysical Properties

**Figure 2:**
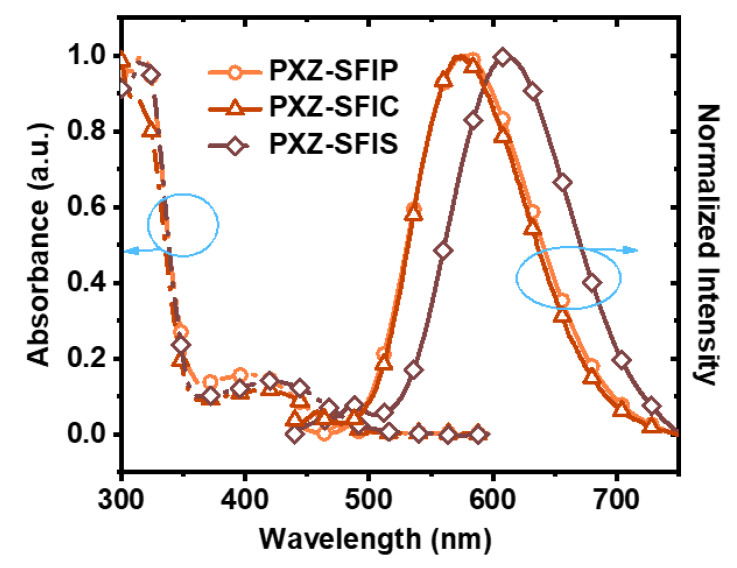
Absorption (Abs.) and emission (Fl.) spectra of **PXZ-SFIP**, **PXZ-SFIC**, and **PXZ-SFIS** (10^−5^ M) measured at room temperature in toluene.

The UV-Vis absorption spectra of the TADF compounds in dilute toluene solutions (10^−5^ mol·L^−1^) are exhibited in Figure 2. The bands below 350 nm with high intensity were ascribed to π-π* absorption from the conjugated skeleton, while the broad structureless bands peaking at 400~430 nm represented characteristic intramolecular charge transfer (ICT) transition from the phenoxazine to dibenzothiophene sulfoximine unit. Upon excitation, the **PXZ-SFIP**, **PXZ-SFIC**, and **PXZ-SFIS** compounds revealed fluorescence with singular Gaussian-shaped emission bands, peaking at 576 nm, 574 nm, and 611 nm, respectively. Amongst three emitters, **PXZ-SFIS,** acquiring more electron-deficient benzenesulfonyl substitution on imine, caused a larger Stocks shift with red-shifted emission, while the emission bands of **PXZ-SFIP** and **PXZ-SFIC** were nearly overlapping with each other. Optical bandgaps of the emitters were 2.60 eV, 2.54 eV, and 2.49 eV, separately, as estimated from the onset of the absorption spectra from the longer wavelength. The decreasing optical bandgap corresponded well with increasing electron deficiency of the functioned dibenzothiophene sulfoximine, giving deepening LUMO levels of −2.59 eV for **PXZ-SFIP**, −2.64 eV for **PXZ-SFIC**, and −2.70 eV for **PXZ-SFIS** (Table 2). 

**Figure 3:**
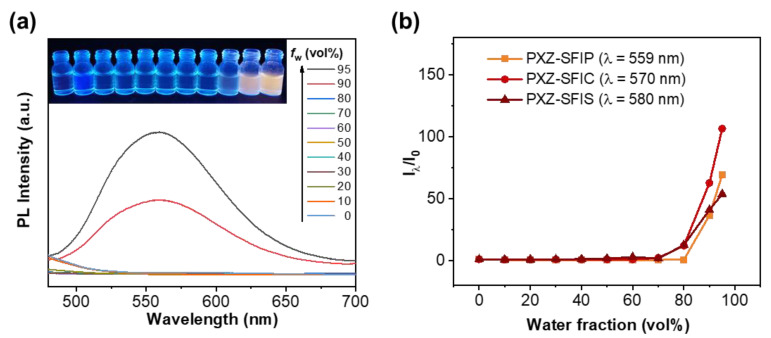
(**a**) PL spectra of **PXZ-SFIP** in THF/water mixtures with different water fractions; inset: photograph of the samples in sequence under excitation of 365 nm. (**b**) Plot of *I*_λ_ nm/*I*_0_ versus water fraction of the solvent mixture, where *I*_0_ is the PL intensity in pure THF solution, *λ* is the peak wavelengths detailed in the legend.

AIE characteristics of the **PXZ-SFIP**, **PXZ-SFIC,** and **PXZ-SFIS** compounds in THF/water mixed solvents with different water fractions (*f*_W_) were investigated, as shown in Figure 3 and Figure S4. In solutions with *f*_W_ lower than 80%, the fully dissolved emitters were almost non-emissive where peripheral functional groups could freely rotate and dissipate energy. Further increasing *f*_W_ led to a significant increase in PL intensities by the formation of aggregates, as explained by the restriction of intramolecular rotation (RIR) of the TADF emitter [45]. This distinct AIE behavior was beneficial for solid-state emission due to the suppression of undesirable aggregation-caused quenching (ACQ), which could potentially improve device performances and alleviate undesirable efficiency roll-off at high brightness in OLEDs.

**Figure 4:**
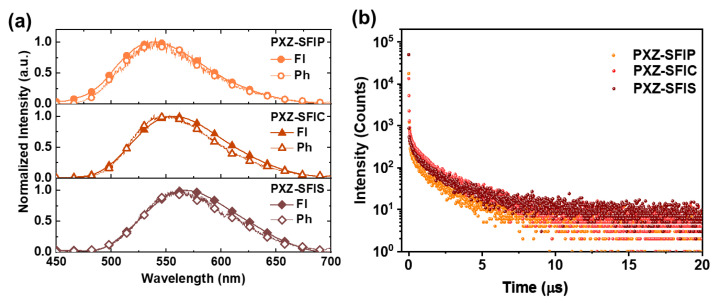
(**a**) Steady-state mission (Fl) spectra and phosphorescence (Ph) spectra of **PXZ-SFIP**, **PXZ-SFIC**, and **PXZ-SFIS** doped in CBP thin film (10 wt%), measured at 77 K. (**b**) Transient PL decay curves of DPEPO thin films doped with 10 wt% of **PXZ-SFIP**, **PXZ-SFIC**, and **PXZ-SFIS** emitters.

Experimental excited state energies were measured by fluorescent and phosphorescent spectroscopy in film state, as an imitation of the environment in emissive layers (EML) of OLEDs. Here, *bis*[2-(diphenylphosphino)phenyl] ether oxide (DPEPO) was selected as host material due to its large energy band gap and high T_1_ level (~3.4 eV). As shown in Figure 4, S_1_ and T_1_ levels of the emitters were estimated from the onset of fluorescent and phosphorescent spectra, giving the values of 2.61/2.59 eV for **PXZ-SFIP**, 2.54/2.52 eV for **PXZ-SFIC**, and 2.51/2.48 eV for **PXZ-SFIS**. The small Δ*E*_ST_s around 0.02~0.03 eV were consistent with the previous theoretical calculation results, assuring an effective up-conversion process from T_1_ to S_1_. To confirm their TADF character, the transient PL decay profiles of the three emitters were recorded in both solution and doped films. The samples in degassed toluene displayed obvious delayed components that could be largely quenched in aerated conditions due to the sensitivity of the triplet state excitons to oxygen (Figure S5). Meanwhile, the transient PL curves of 10 wt% doped DPEPO films could also be fitted with biexponential functions, with prompt and delayed fluorescence lifetimes (*τ*_p_/*τ*_d_) of 30.5 ns/1.2 μs, 28.7 ns/1.3 μs, and 29.9 ns/1.2 μs for **PXZ-SFIP**, **PXZ-SFIC**, and **PXZ-SFIS**, separately, and the ratios of the delayed fluorescence were 64.4%, 62.3%, and 61.3%. The absolute photoluminescent quantum yield (*Φ*_PL_) of the DPEPO films doped with the TADF emitters was found to be 52%/41%/21% for **PXZ-SFIP**/**PXZ-SFIC**/**PXZ-SFIS**. 

Subsequently, the S_1_→S_0_ radiative and nonradiative decay rate constants (*k*_r,S_, *k*_nr,S_) together with the ISC (*k*_ISC_) and RISC rate constants (*k*_RISC_) were extrapolated to provide more in-depth information on the TADF characteristics and PL efficiencies (Table 3). The emitters shared comparable *k*_ISC_ and *k*_RISC_ values due to similar Δ*E*_ST_s, noting that all *k*_RISC_s were at the state-of-the-art level of ~10^6^ s^−1^ to guarantee effective utilization of triplet excitons. On the contrary, the *k*_r,S_ values decreased significantly from 6.1/5.4 × 10^6^ s^−1^ of **PXZ-SFIP**/**PXZ-SFIC** to 2.7 × 10^6^ s^−1^ of PXZ-SFIS, in line with the trend of theoretically predicted oscillator strengths. The **PXZ-SFIS** also possessed the largest *k*_nr,S_ in these emitters, with this value even outcompeting its *k*_r,S_, manifesting considerable nonradiative dissipation of excitons through internal conversion with its more stabilized CT energy as governed by the energy-gap law.

### 3.5. Device Characterization

**Figure 5:**
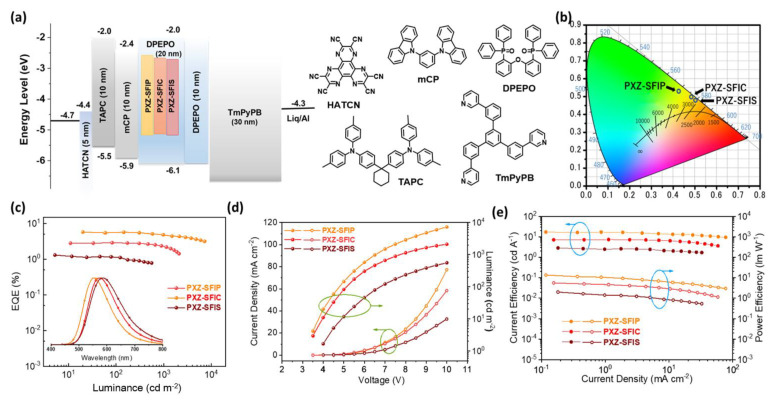
(**a**) Energy level alignment of all the materials used in the current study (**left**) and schematic diagrams of the basic structures of the quadruple-layer device (**right**). (**b**) The Commission Internationale de I’Eclairage coordinates recorded at maximum EQE (**c**) External quantum efficiency (EQE) versus current density (J); inset: normalized EL spectra of the devices. (**d**) Current density–voltage (J–V) and luminance–voltage (L–V) characteristics of the devices. (**e**) Power efficiency (PE) and current efficiency (CE) versus current density (J).

Electroluminescent properties of the TADF emitters were further evaluated by OLEDs with the TADF emitter doped films as the light-emitting layer. The devices adopted the configuration of ITO/HATCN (5 nm)/TAPC (10 nm)/mCP (10 nm)/DPEPO: 10 wt% TADF emitter (20 nm)/DPEPO (10 nm)/Liq (1.5 nm)/Al (100 nm) (Figure 5a). Here, 1,4,5,8,9,11-hexaazatriphenylenehexacarbonitrile (HATCN) and 8-hydroxyquinolinolato-lithium (Liq) were used as the hole injection layer (HIL) and electron injection layer (EIL), respectively. 1,1-*Bis*[(di-4-tolylamino)phenyl]cyclohexane (TAPC) and 1,3,5-*Tris*(3-pyridyl-3-phenyl)benzene (TmPyPB) served as the hole transport layer (HTL) and electron-transporting layer (ETL), respectively. 1,3-Di(9H-carbazol-9-yl)benzene (mCP) and DPEPO were applied as exciton-blocking layers (EBL). DPEPO was selected as the host material in the emitting layer (EML) with its sufficiently high T_1_ energy to avoid energy back transfer. 

In 10 wt% doped devices, the EL spectra clearly originated from the TADF emitters, suggesting complete energy transfer from DPEPO host to dopants. The devices displayed yellow to orange emissions peaking at 553 nm for **PXZ-SFIP**, 572 nm for **PXZ-SFIC**, and 584 nm **PXZ-SFIS**, respectively. **PXZ-SFIP** with the highest *Φ*_PL_ exhibited the best device performance with the maximum external quantum efficiency (EQE_max_) of 5.75%, current efficiency (CE_max_) of 17.06 cd·A^−1^, and power efficiency (PE_max_) of 13.40 lm·W^−1^. Comparatively, devices based on **PXZ-SFIC** and **PXZ-SFIS** showed slightly inferior efficiencies with maximum EQE values at 3.17% and 1.30%, separately, which could be well explained by their suppressed singlet radiative transition and faster non-radiative internal conversion process. It is noteworthy that the devices did not come across serious efficiency roll-off at high brightness, attributing to the emitters’ AIE character, i.e., the efficiency roll-off value of the **PXZ-SFIP** device was only 16.7% at 1000 cd·m^−2^. Additionally, the shortened *τ*_DF_s and efficient RISC process may also suppress notorious triplet-triplet annihilation (TTA), singlet-triplet annihilation (STA), or triplet polaron exciton annihilation. The EL data for all the emitters are summarized in Table 4. 

## 4. Conclusions

In summary, a series of TADF emitters were constructed by 10*H*-phenoxazine and novel dibenzothiophene sulfoximine derivatives. By attaching diphenylphosphoryl, benzoyl, and benzenesulfonyl units with gradually increased electron deficiencies at the secondary amine in dibenzothiophene sulfoximine acceptors, bathochromic shifted fluorescence from yellow to orange were obtained with more stabilized CT energy. The orthogonal D-A conformation endowed these emitters nearly degenerate S_1_ and T_1_ state, high *k*_RISC_s at the level of ~10^6^ s^−1^, and efficient delayed fluorescence. At the same time, the emitters manifested distinct AIE properties with *Φ*_PL_ up to 52% in film state. Subsequently, devices based on **PXZ-SFIP** as emitting material realized a maximum EQE of 5.8% with reduced efficiency roll-off. This work not only demonstrates dibenzothiophene sulfoximine as potential construction units for AIE-active TADF emitters, but also provides a meaningful strategy for designing color-managing acceptors.

## Figures and Tables

**Table 1:** Calculated photophysical parameters of the designed emitters.

Entry	LUMO	HOMO	*E*_g_	S_1_	T_1_	Δ*E*_ST_
	(eV)	(eV)	(eV)	(eV)	(eV)	(eV)
**PXZ-SFIP**	−2.23	−4.97	2.74	2.38	2.29	0.08
**PXZ-SFIC**	−2.39	−5.08	2.69	2.32	2.29	0.03
**PXZ-SFIS**	−2.58	−5.26	2.68	2.29	2.26	0.02

**Table 2:** Photophysical properties of the TADF emitters.

Compound	λ_abs_ ^a^ (nm)	λ_em_ ^a^ (nm)	λ_em_ ^b^ (nm)	*E*_g_ ^c^ (eV)	HOMO ^d^/LUMO ^e^ (eV)	S_1_ ^f^/T_1_ ^g^ (eV)	Δ*E*_ST_ (eV)
**PXZ-SFIP**	317/403	576	539	2.60	−5.19/−2.59	2.61/2.59	0.02
**PXZ-SFIC**	313/413	574	556	2.54	−5.18/−2.64	2.54/2.52	0.02
**PXZ-SFIS**	318/421	611	568	2.49	−5.19/−2.70	2.51/2.48	0.03

^a^ Measured in toluene solution (10^−5^ M) at room temperature; ^b^ Measured in the doped films with TADF emitters in CBP host (10 wt%); ^c^ Estimated by the onset of the UV-vis spectra from longer wavelengths; ^d^ Determined by the onset of the oxidation potentials and ^e^ HOMO minus optical energy gaps calculated from the onset of UV-Vis spectra. ^f^ Estimated from the onset of low-temperature fluorescent and ^g^ phosphorescent spectra of doped films with TADF emitters in CBP host (10 wt%).

**Table 3:** Photophysical parameters of 10 wt% doped DPEPO films of the TADF compounds.

Compound	*Φ*_PL_	*τ*_PF_	*τ*_DF_	*k*_r,S_	*k*_nr,S_	*k*_ISC_	*k*_RISC_
	(%)	(ns)	(μs)	(×10^6^ s^−1^)	(×10^6^ s^−1^)	(×10^7^ s^−1^)	(×10^6^ s^−1^)
**PXZ-SFIP**	52.0	30.5	1.2	6.1	5.6	2.1	2.4
**PXZ-SFIC**	41.0	28.7	1.3	5.4	7.8	2.2	2.1
**PXZ-SFIS**	21.0	29.9	1.2	2.7	10.2	2.1	2.2

**Table 4:** Summary of device performances.

Emitter	*V*_on_ ^a^ (V)	CE_max_ ^b^ (cd A^−1^)	PE_max_ ^b^ (lm W^−1^)	EQE ^b^ (%)	EL_peak_ (nm)	CIE ^c^ (x,y)
**PXZ-SFIP**	3.5	17.06	13.40	5.75/5.58/4.79	553	(0.42, 0.54)
**PXZ-SFIC**	3.5	8.01	7.19	3.17/2.89/2.27	572	(0.48, 0.50)
**PXZ-SFIS**	4.0	2.85	1.99	1.30/1.14/-	584	(0.50, 0.48)

^a^ Turn-on voltage (at a brightness of 1 cd·m^−2^); ^b^ the maximum CE (current efficiency), the maximum PE (power efficiency); EQE (external quantum efficiency): maximum, values at 100 cd·m^−2^, and 1000 cd·m^−2^; ^c^ CIE: Commission Internationale de l’Eclairage chromaticity coordinates.

## Data Availability

Data on the compounds are available from the authors.

## Supplementary Materials

The following are available online, Figure S1: The thermogravimetric analysis (TGA) plots of PXZ-SFIS, PXZ-SFIC and PXZ-SFIP under N_2_ stream (flow rate: 20 mL min^−1^; heating rate: 10 °C min^−1^), Figure S2: DSC thermograms (second heating cycle) of **PXZ-SFIS**, **PXZ-SFIC** and **PXZ-SFIP** under N_2_ stream (flow rate: 20 mL min^−1^; heating rate: 10 °C min^−1^), Figure S3: Cyclic voltammograms of **PXZ-SFIP**, **PXZ-SFIC**, and **PXZ-SFIS** in 1 × 10^−3^ M dichloromethane, Figure S4: PL spectra of (a) **PXZ-SFIC** and (b) **PXZ-SFIS** in THF/H_2_O mixed solvents with different water volume fractions, Figure S5: Transient PL decay curves of **PXZ-SFIP**, **PXZ-SFIC**, and **PXZ-SFIS** in 1 × 10^−5^ M toluene solution, Table S1: Natural transition orbitals (NTO) in the S_1_ and T_1_ states of the TADF emitters, Table S2: Thermal analysis of the three compounds.

## Sample Availability

Samples of the compounds are available from the authors.
