# Supplementary material for: Phenoxazine-Dibenzothiophene Sulfoximine Emitters Featuring Both Thermally Activated Delayed Fluorescence and Aggregation Induced Emission
Source: Molecules. 2021 Aug 29;26(17):5243. doi: 10.3390/molecules26175243 (PMC8434553; doi:10.3390/molecules26175243)
Supplement: Supplementary file 1 [file molecules-26-05243-s001.zip › molecules-1343283-supplementary.pdf]

Supporting information

Phenoxazine-dibenzothiophene sulfoximine emitters featuring both thermally activated delayed fluorescence and aggregation induced emission

Yiyu Yang,[a,b] Ran Xiao,[a,b] Xiaosong Cao,[a,] Zhanxiang Chen,[c] Xialei Lv,[a] Youming Zhang,[a]* Shaolong Gong,[c] Yang Zou,[a] and Chuluo Yang[a,c]*

Corresponding authors

*xcao@szu.edu.cn

*zhangyouming1218@126.com

Table of Contents

Figure S1. The thermogravimetric analysis (TGA) plots of PXZ-SFIS, PXZ-SFIC and PXZ-SFIP under N_2 stream (flow rate: 20 mL min^{-1}; heating rate: 10 °C min^{-1})......3

Figure S2. DSC thermograms (second heating cycle) of **PXZ-SFIS**, **PXZ-SFIC** and **PXZ-SFIP** under N_2 stream (flow rate: 20 mL min^{-1}; heating rate: 10 °C min^{-1}). 3

Figure S3. Cyclic voltammograms of **PXZ-SFIP**, **PXZ-SFIC**, and **PXZ-SFIS** in 1×10^{-3} M dichloromethane......4

Figure S4. PL spectra of (a) **PXZ-SFIC** and (b) **PXZ-SFIS** in THF/H_2O mixed solvents with different water volume fractions......4

Figure S5. Transient PL decay curves of **PXZ-SFIP**, **PXZ-SFIC**, and **PXZ-SFIS** in 1×10^{-5} M toluene solution......5

Table S1. Natural transition orbitals (NTO) in the S_1 and T_1 states of the TADF emitters......2

Table S2. Thermal analysis of the three compounds......3

Theoretical Calculation

Table S1. Natural transition orbitals (NTO) in the S_1 and T_1 states of the TADF emitters.

Emitter	State	*Hole*	*Particle*
**PXZ-SFIP**	S_1	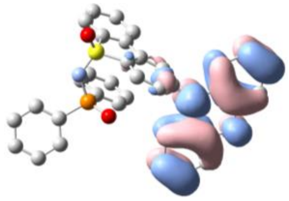 f=0.0177	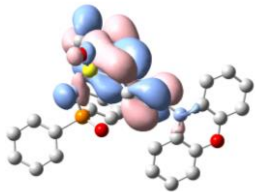
	T_1	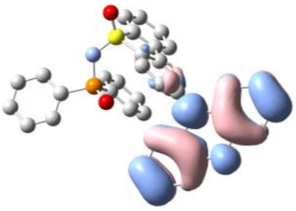 f=0.0000	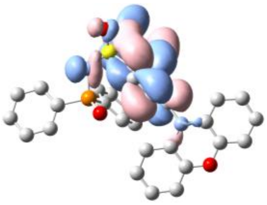
**PXZ-SFIC**	S_1	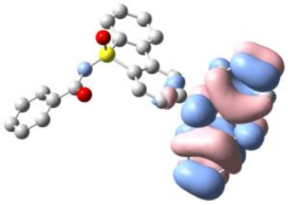 f=0.0028	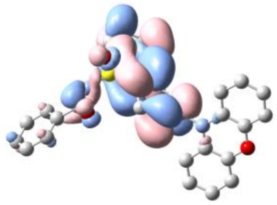
	T_1	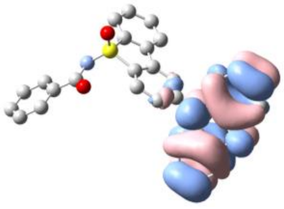 f=0.0000	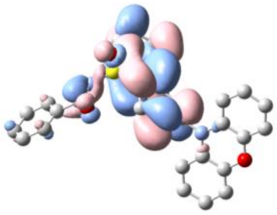
**PXZ-SFIS**	S_1	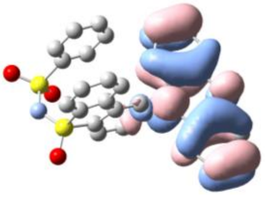 f=0.0001	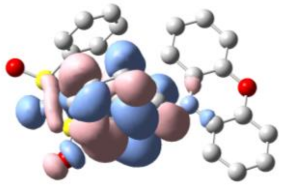
	T_1	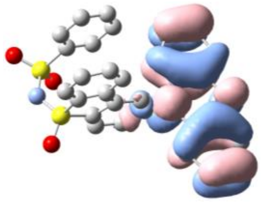 f=0.0000	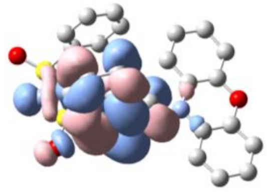

Thermal Properties

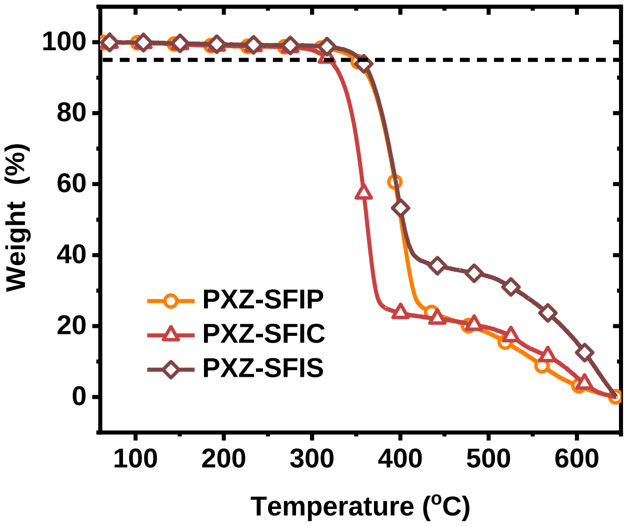

Figure S1. The thermogravimetric analysis (TGA) plots of PXZ-SFIS, PXZ-SFIC and PXZ-SFIP under N_2 stream (flow rate: 20 mL min^{-1}; heating rate: 10 °C min^{-1}).

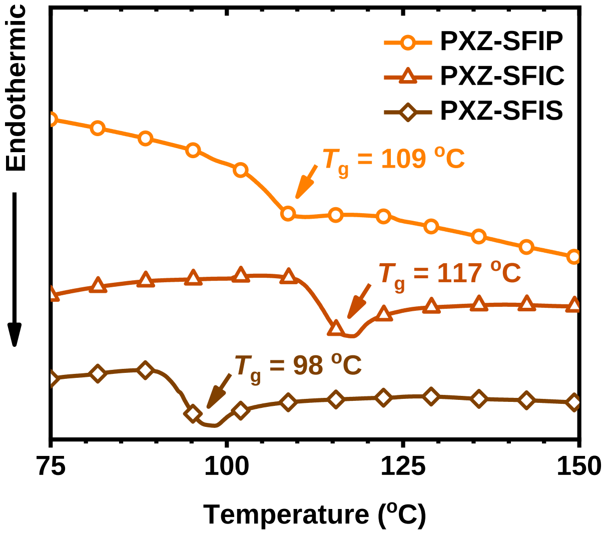

Figure S2. DSC thermograms (second heating cycle) of **PXZ-SFIS**, **PXZ-SFIC** and **PXZ-SFIP** under N_2 stream (flow rate: 20 mL min^{-1}; heating rate: 10 °C min^{-1}).

Table S2. Thermal analysis of the three compounds.

Compound	T_g[a)] (°C)	T_d[b)] (°C)
**PXZ-SFIP**	109	350
**PXZ-SFIC**	117	320
**PXZ-SFIS**	98	355

Electrochemical Properties

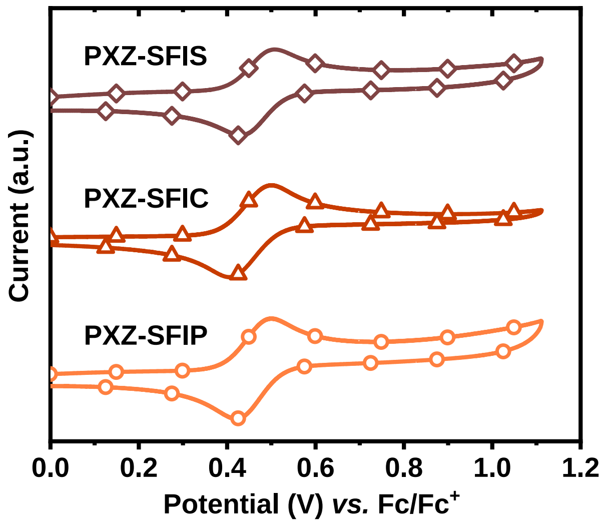

Figure S3. Cyclic voltammograms of **PXZ-SFIP**, **PXZ-SFIC**, and **PXZ-SFIS** in 1×10^{-3} M dichloromethane.

Photophysical Properties

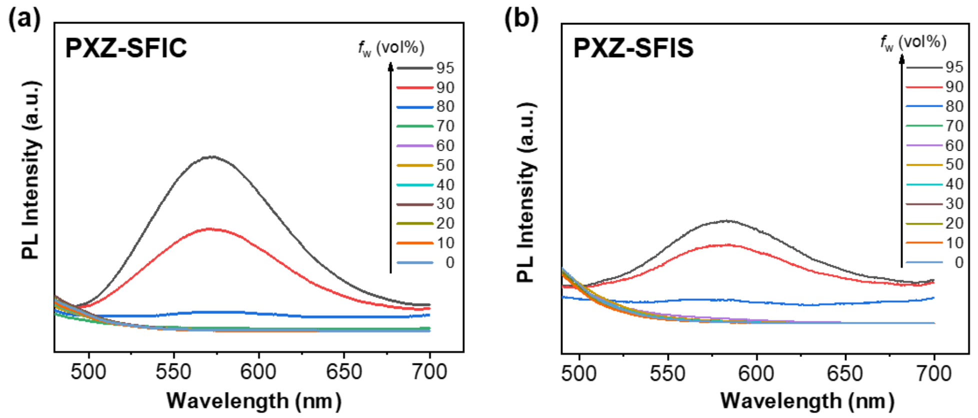

Figure S4. PL spectra of (a) **PXZ-SFIC** and (b) **PXZ-SFIS** in THF/H_2O mixed solvents with different water volume fractions.

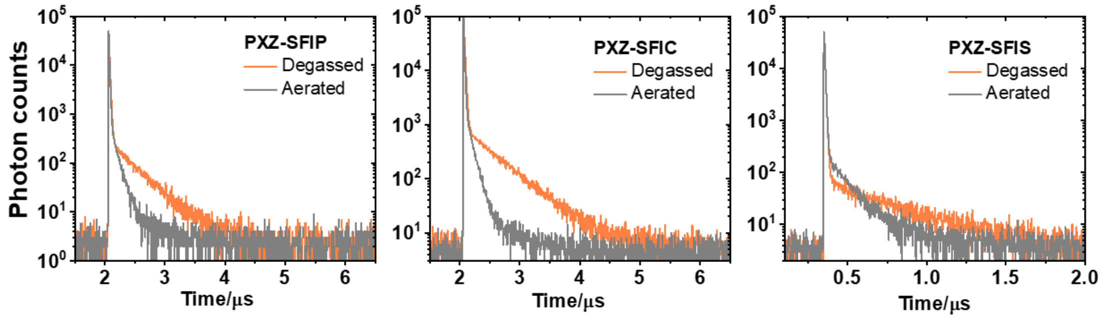

Figure S5. Transient PL decay curves of **PXZ-SFIP**, **PXZ-SFIC**, and **PXZ-SFIS** in 1×10^{-5} M toluene solution.

Calculations of Rate Constants

The rate constants of radiative decay ($k_{r,S}$) and nonradiative decay ($k_{nr,S}$) from S_1 to S_0 states, the rate constants of intersystem crossing (k_{ISC}) and reverse intersystem crossing (k_{RISC}) could be estimated using the following equations: [1, 2]

$$k_{r,S} = \Phi_p k_p + \Phi_d k_d \approx \Phi_p k_p \quad \text{Eq.(1)}$$

where Φ_p and Φ_d indicate prompt and delayed fluorescence components and can be distinguished from the total Φ_{PL} by comparing the integrated intensities of prompt and delayed components in the transient PL spectra; whereas k_p and k_d represent the decay rate constants for prompt and delayed fluorescence, respectively, which are determined experimentally from the decay fluorescence time constants (τ_p and τ_d, addressed in the main text).

$$k_p = \frac{1}{\tau_p} \quad \text{Eq.(2)}$$

$$k_d = \frac{1}{\tau_d} \quad \text{Eq.(3)}$$

$$k_{nr,S} = \frac{1-\Phi_{PL}}{\Phi_{PL}} k_{r,S} \quad \text{Eq.(4)}$$

$$k_{ISC} = k_p - k_{r,S} - k_{nr,S} \quad \text{Eq.(5)}$$

$$k_{RISC} = (k_p k_d \Phi_d)/(k_{ISC}\Phi_p) \quad \text{Eq.(6)}$$

NMR Spectra

^{1}H NMR of **PXZ-SFIP**

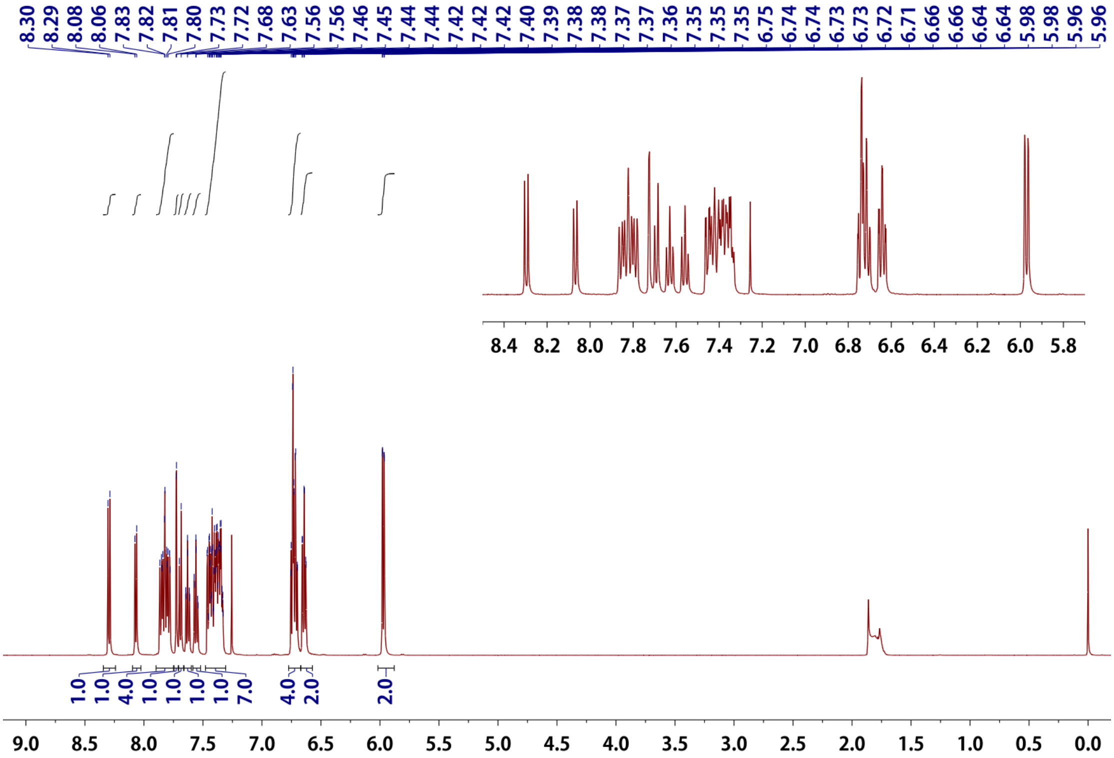

^{13}C NMR of **PXZ-SFIP**

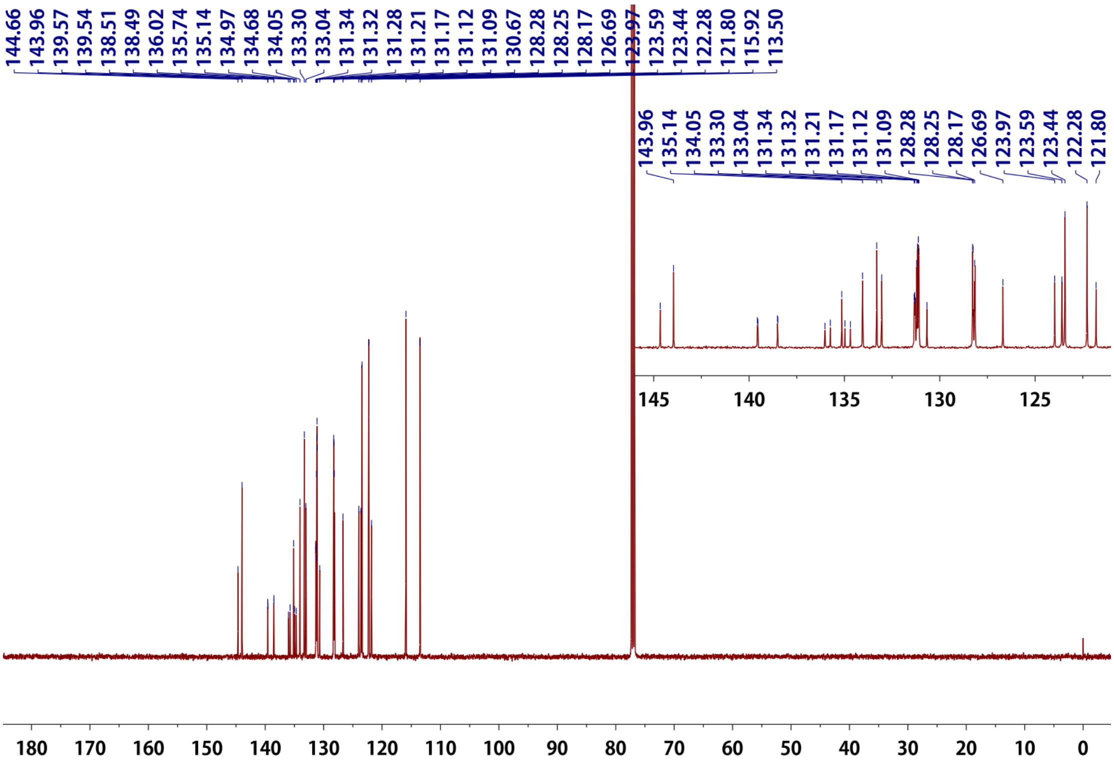

^{1}H NMR of **PXZ-SFIC**

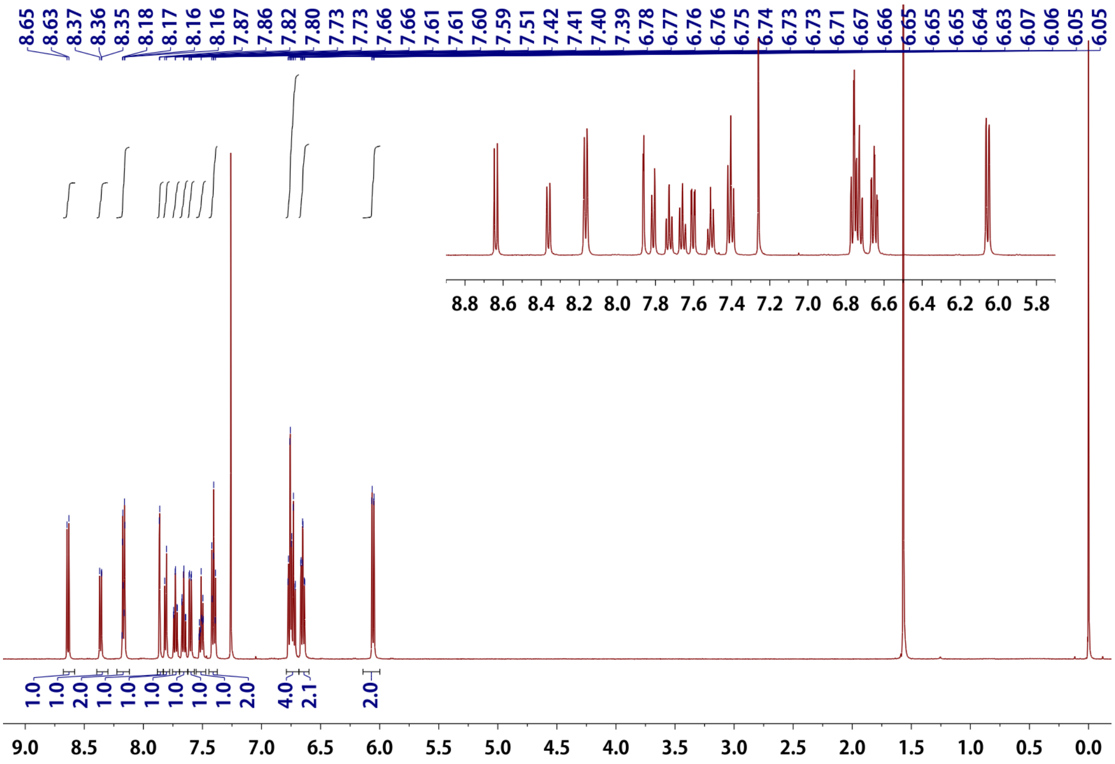

^{13}C NMR of **PXZ-SFIC**

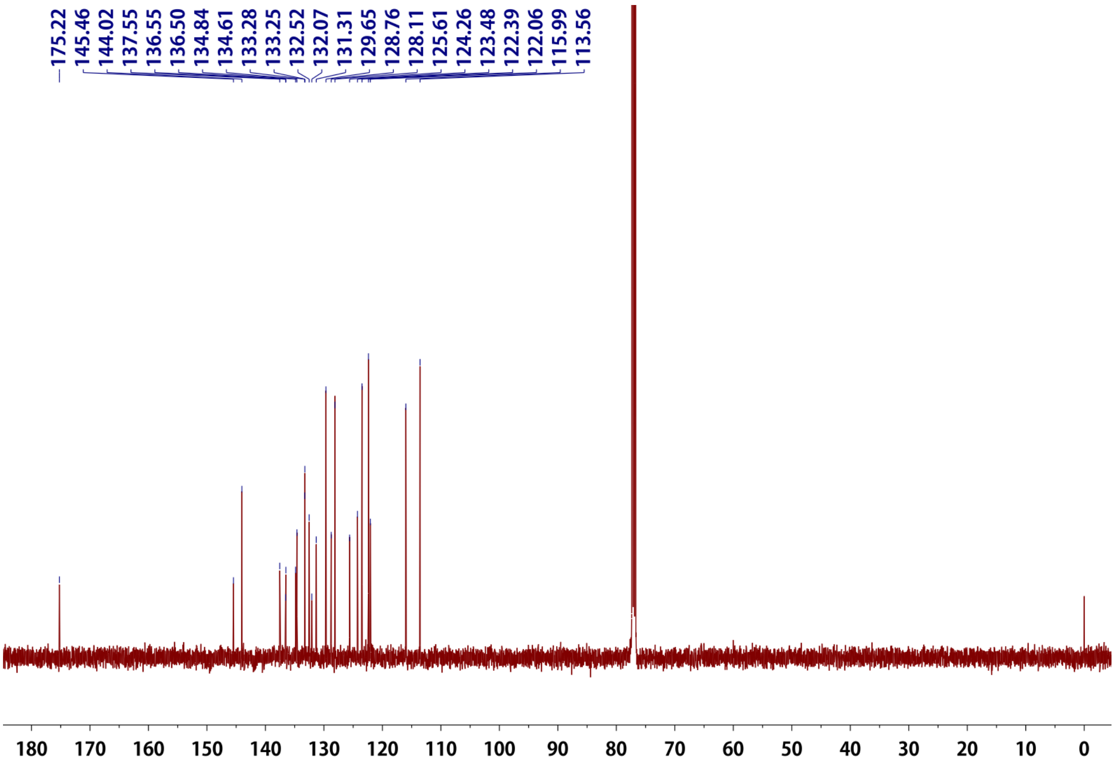

^{1}H NMR of **PXZ-SFIS**

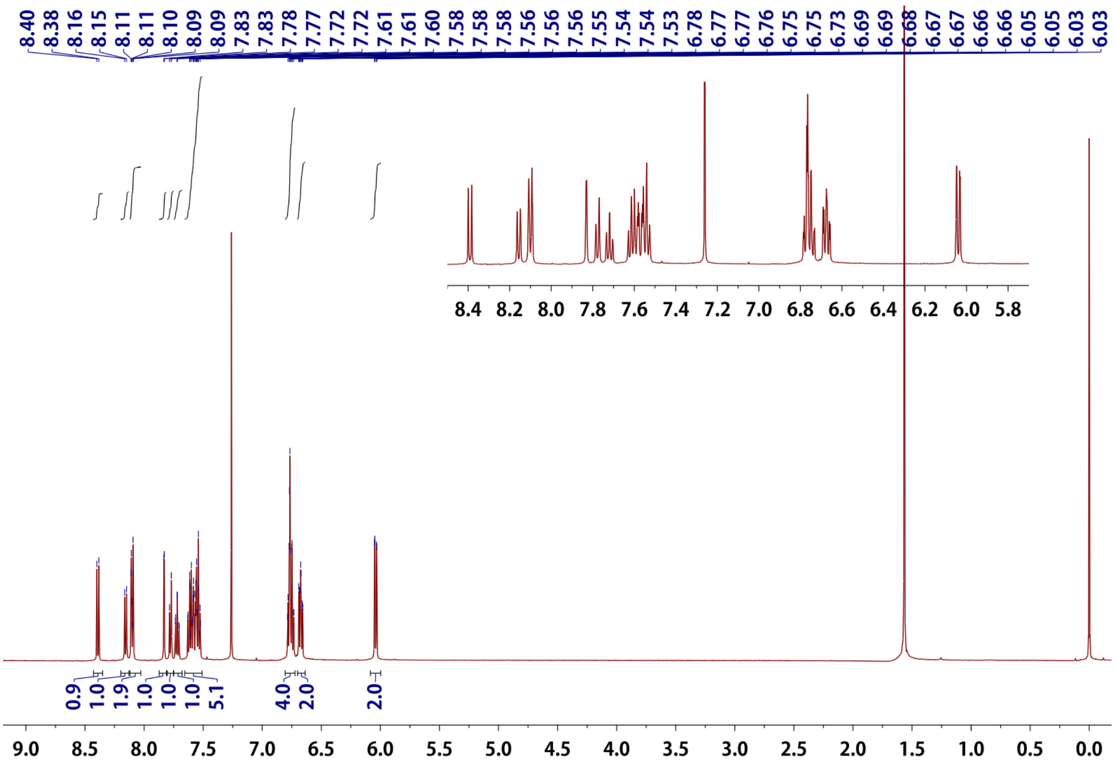

^{13}C NMR of **PXZ-SFIS**

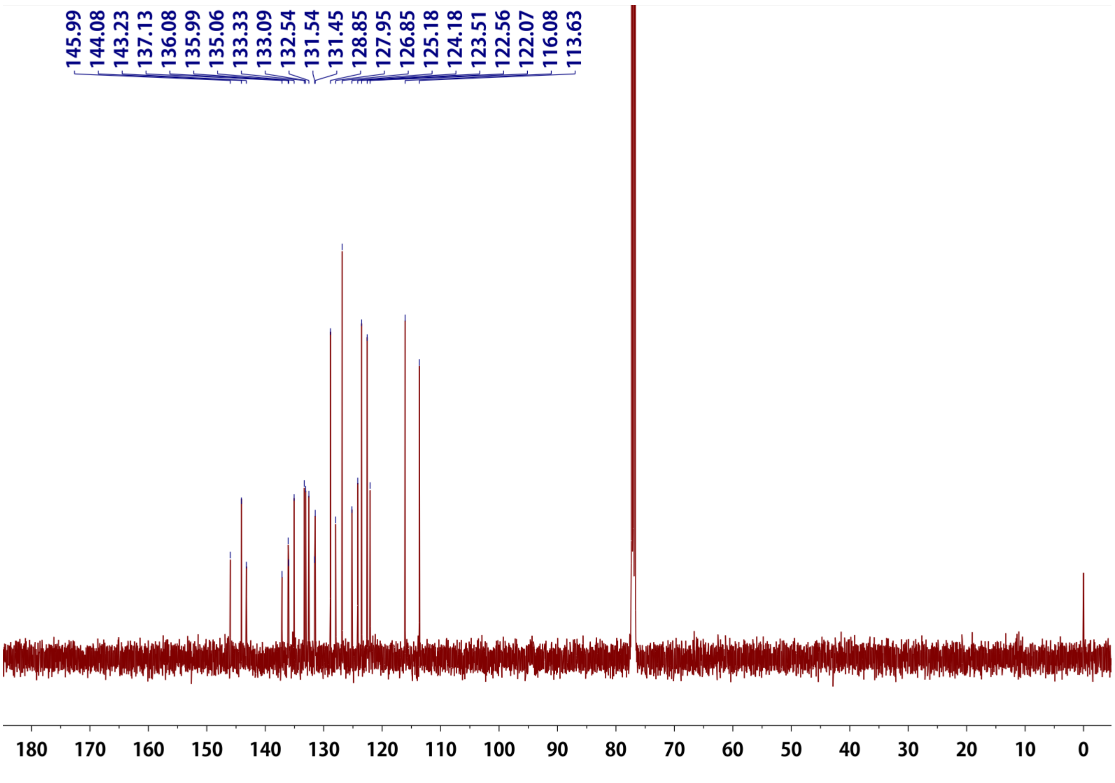

References

1. Pan, K.-C.; Li, S.-W.; Ho, Y.-Y.; Shiu, Y.-J.; Tsai, W.-L.; Jiao, M.; Lee, W.-K.; Wu, C.-C.; Chung, C.-L.; Chatterjee, T.; Li, Y.-S.; Wong, K.-T.; Hu, H.-C.; Chen, C.-C.; Lee, M.-T., Efficient and Tunable Thermally Activated Delayed Fluorescence Emitters Having Orientation-Adjustable CN-Substituted Pyridine and Pyrimidine Acceptor Units. *Adv. Funct. Mater.* **2016,** 26, (42), 7560-7571.
2. Zhang, Q.; Kuwabara, H.; Potscavage, W. J.; Huang, S.; Hatae, Y.; Shibata, T.; Adachi, C., Anthraquinone-Based Intramolecular Charge-Transfer Compounds: Computational Molecular Design, Thermally Activated Delayed Fluorescence, and Highly Efficient Red Electroluminescence. *J. Am. Chem. Soc.* **2014,** 136, (52), 18070-18081.
